# The Use of Subscores in Higher Education: When Is This Useful?

**DOI:** 10.3389/fpsyg.2017.00305

**Published:** 2017-03-07

**Authors:** Rob R. Meijer, Anja J. Boevé, Jorge N. Tendeiro, Roel J. Bosker, Casper J. Albers

**Affiliations:** ^1^Faculty of Behavioral and Social Sciences, Psychometrics and Statistics, University of Groningen Groningen, Netherlands; ^2^Faculty of Behavioral and Social Sciences, Education, University of Groningen Groningen, Netherlands

**Keywords:** classroom testing, diagnostic testing, formative feedback, test format, subscores, validity open-ended questions

## Abstract

Assessment in higher education is challenging because teachers face more students, with less contact time as compared to primary and secondary education. Therefore, teachers and management are often interested in efficient ways of giving students diagnostic feedback and providing information on the basis of subscores is one method that is often used in large-scale standardized testing. In this article we discuss some recent psychometric literature that warns against the use of subscores in addition to the use of total scores. We illustrate how the added value of subscores can be evaluated using two college exams: A multiple choice exam and a combined open-ended question and multiple choice exam; these formats are often used in higher education and represent cases in which using subscores may be informative. We discuss the implications of our findings for future classroom evaluation.

## Introduction

For teachers in higher education, student assessment through administering and scoring exams is a common and efficient method to test large groups of students. Cizek (2009) defined a test (or exam) as “a systematic sample of a person’s knowledge, skill, or ability” and assessment as a much broader planned process of gathering such information for different purposes. Assessment in higher education is challenging for teachers since they face more students, with less contact-time compared to teachers in primary and secondary education. Using a single test for multiple purposes in assessment is, therefore, an efficient way of assessment. Providing students with feedback is often suggested to improve the quality of learning, and thereby increasing student performance (Black and Wiliam, 1998, 2003). One way to provide feedback while keeping teacher burden low, is to report subscores, that is, to report the sum of item scores on a specified number of items, because it is assumed that these subscores may provide additional information to the total score on the exam. This idea is not new and there are many examples where subscores on exams or tests are used for diagnostic, formative, and remedial purposes (Ketterlin-Geller and Yovanoff, 2009; Schneider and Andrade, 2013; Harks et al., 2014). For example, the total score on a reading comprehension test may be reported together with subscores that reflect specific reading skills, like being able to understand the meaning of a story as opposed to being able to read and understand individual sentences (Reckase and Xu, 2014).

In large-scale testing, reporting subscores is sometimes even required. For example, in the U.S. for some educational programs it is required that “students should receive diagnostic reports that allow teachers to address their specific academic need; subscores could be used in such a diagnostic report” (Sinharay, 2010, p. 150). In primary education in the Netherlands the use of subscores for different topics like reading comprehension and arithmetic is required for the general test that helps to determine which type of secondary education students will follow (Rijksoverheid, 2016).

Before reporting subscores, teachers and instructors should provide evidence that observed subscores contain unique information over and above the observed total score in terms of the true subscores. In the often cited Standard 1.14 of the Standards for Educational and Psychological Testing (American Educational Research Association, American Psychological Association, and National Council on Measurement in Education, 2014) it is said that “When interpretation of subscores, score differences, or profiles is suggested, the rationale and relevant evidence in support of such interpretation should be provided” and “When a test provides more than one score, the distinctiveness and reliability of the separate scores should be demonstrated, and the interrelationship of those scores should be shown to be consistent with the construct(s) being measured” (p. 27). Like incorrect or invalid test scores may have serious detrimental effects on grading or selection, unreliable and invalid subscores may have detrimental effects on decisions made to assign students to remedial teaching groups or to invest more time in particular knowledge domains.

For commercial tests and questionnaires, techniques like factor analysis and scale analyses are often used to investigate whether it is useful to distinguish separate clusters of item scores. Because users of large-scale tests are expected to justify the interpretation of subscores, the relevance of investigating the quality of subscores is clear in this context. It is therefore also not surprising that recent psychometric studies in large-scale educational testing (e.g., Sinharay et al., 2011) discussed when subscores provide additional information to the total scores. However, many of these studies are rather technical and aimed at educational researchers. As a result, these papers are often difficult to understand for practitioners.

As Cizek (2009) argued, however, the context of classroom assessment is different from the context of large-scale assessment. The rigorous and extensive test-development techniques of large-scale tests are not generally used for classroom tests. An important reason for the latter is that, in general, stakes are lower in classroom testing than in large-scale testing. In higher education, however, tests results sometimes determine whether a student can follow another course or will suffer financial consequences from study delay. If any information on item and test quality is given to or monitored by teachers in higher education, these are generally classical indices like proportion-correct scores, item-total correlations, and reliability estimates. As we will demonstrate below, the classical test-theory framework can also be used to evaluate the quality of subscores in classroom tests in higher education.

In this paper we analyzed two exams from a psychology program with a method that can be used to investigate whether subscores have added value over and above total scores. Using this method, the usefulness of reporting subscores for different tests used in higher education will be enhanced. We used both a multiple-choice exam and an exam that consisted both of multiple-choice items and open-ended questions. For this latter exam we also investigated the added value of the open-ended questions to the multiple-choice questions in terms of measurement precision.

This paper has the following structure. We first discuss an existing method that can be used to investigate whether subscores have added value. Second, we analyzed the college exams. Finally, we discuss the implications of our study for formative assessment in psychology education. In this paper we use psychometric arguments; every teacher or instructor is, of course, free to decide that information obtained from subscores is still useful irrespective of the outcome of a psychometric analysis. However, we think that it may be illuminating to see that information obtained from subscores that seems intuitively useful may not contain additional information over and above the total score.

### Rationale behind the Added Value of Subscores and Findings in the Literature

We used a method discussed by Haberman (2008). Assume that we have an exam and that we calculate the total score on this exam as the number of questions answered correctly. Furthermore, assume that we are interested in reporting subscores on subsets of items. Haberman’s method (to be discussed in more detail below) is based on two important psychometric indicators to determine whether or not subscores may have added value to the total score. The first is the correlation between the (true) subscore and the total score and the second is the estimated reliability of the total score and the individual subscores. The idea is that when the reliability of the individual subscores is relatively low, often due to a limited number of items, and the correlation between subscores and the total score is relatively high, reporting subscores in addition to reporting the total score has no *added* value over and above reporting only the total score.

Sinharay (2010) reviewed a number of large-scale exams administered in the U.S. and concluded that “subscores on operational tests have more often been found not to be useful than to be useful.” He also noted that “there is a lack of studies that demonstrated the validity of inferences made from subscores.” For example, there is lack of evidence that subscores are related to other external criteria and that the incremental validity of subscores is valuable when subtest scores are highly related. Based on empirical and simulation studies Sinharay (2010) concluded that:

(a) Subscores based on tests smaller than 10 items almost never have added value because the reliability of these subscores is often too low, and that
(b) “The most important finding is that it is not easy to have subscores that have added value. Based on the results here, the subscores have to consist of at least about 20 items and have to be sufficiently distinct from each other to have any hope of having added value. Several practitioners believe that subscores consisting of a few items may have added value if they are sufficiently distinct from each other. However, the results in this study provide evidence that is contrary to that belief. Subscores with 10 items were not of any added value even for a realistically extreme (low) disattenuated correlation of 0.7.”

However, these rules-of-thumb were predominantly obtained from large-scale exams and it is unclear whether these results can be generalized when investigating the added value for classroom tests.

### Method Proposed by Haberman (2008)

As discussed above, in the present study we concentrated on a method suggested by Haberman (2008) that is based on classical test theory. Many tests used in higher education are evaluated using classical test theory indices and so this method can be easily applied in this context. To determine whether subscores have added value over and above the total score, Haberman (2008) used the proportional reduction in mean squared error (PRMSE). The central idea is that one should only use a subtest score over a total score when it can be shown that the observed subtest score leads to a larger reduction in mean squared error in estimating the true subtest score than the observed total score. It can be shown that this is the case when the correlation between the true subtest score and the observed subtest score is larger than the correlation between the true subtest score and the observed total score (Haberman, 2008). The larger the PRMSE, the smaller the mean squared error to estimate the true subscore.

Let PRMSE_s_ denote the PRMSE associated with the regression estimate of the true subscore on subtest *s* by means of the observed subscore on subtest *s*. Let PRMSE_x_ denote the PRMSE associated with the regression estimate of the true subscore on subtest *s* by means of the observed total score on test *x*. Haberman (2008) showed that PRMSE_s_ equals the estimated reliability of the observed subscore. The idea is that the observed subscore provides added value over and above the observed total score to estimate the true subscore when the observed reliability of the subtest score (PRMSE_s_) is larger than PRMSE_x_. In the context of typical performance testing in psychology, Reise et al. (2013) give a step-by-step instruction on how to calculate the PRMSE_x_.

## Materials and Methods

We investigated the added value of subscores on two exams from a degree program in psychology. The exams were taken by second year bachelor’s degree students at an international degree program in psychology at a Dutch University, in the academic year 2014–2015. Exam records were collected primarily for educational purposes and these existing data are allowed to be used for research purposes in accordance with this university’s privacy policy.

The first exam (34 items) was from a course on Test Theory taken by 319 students. We chose to split the exam into two subtests, namely 14 items that could be classified as factual knowledge and 20 items that reflected conceptual understanding of test construction and test use. These subtests were classified after test-construction and in a subjective manner by the authors of this study. In the faculty where this research took place, there was an interest in using Bloom’s taxonomy (Krathwohl, 2002) to give students feedback on the depth of their understanding. This inspired investigating whether classifying a test used in practice into subtests based on different types of knowledge would lead to subtest scores that provided more information than the total score.

The second exam was from a statistics course and consisted of 5 short-answer/partial credit open-ended questions and 20 multiple choice questions, where the final grade was computed based on 25% of the score on the open-ended questions and on 75% of the score on the multiple choice questions. The exam was administered to 350 examinees that followed the course in the English language. For the open-ended part of the exam, a grade between 1 and 10 was assigned.

There is a large body of literature that shows that, in general, administering multiple-choice questions is a more efficient way of measuring knowledge than open-ended questions and that open-ended questions are not superior to multiple choice items in terms of reliability and validity (e.g., Hift, 2014). However, both teachers and students are sometimes in favor of open-ended questions. One of the main reasons for teachers to use open-ended questions is that teachers are interested in students’ reasoning, to see what students know and what they do not know so that they can use this knowledge in future lectures. Also, teachers would like to see that students could perform certain operations that are more difficult to measure using multiple-choice items. Furthermore, students are sometimes in favor of open-ended questions because they have the feeling that these questions better reflect what they know.

On the Statistics exam, subscores of the open-ended questions and subscores of the multiple-choice questions were reported to students during the inspection of the exam results. Although the teachers did not provide further diagnostic information from these subscores, it is not unreasonable to take the next step and to consider whether these subscores provide added value such that students may use information from the subscores to determine their study strategy for a possible re-sit exam. We used the function prmse.subscores.scales from the *R* package sirt (Robitzsch, 2016) to calculate these PRMSE’s.

## Results

For both exams we calculated the PRMSE_s_ and the PRMSE_x_. The PRMSE_s_ equals the reliability of the observed subscore. As discussed above, the subscores provided added value over the total score if and only if PRMSE_s_ is larger than PRMSE_x_. For both tests, the subscores did not provide added value over the total score. Below we discuss the results for each exam in more detail.

For the Test Theory exam, with a total test reliability of 0.71, the observed subtest score reliabilities (PRMSE_s_) equaled 0.58 for the conceptual understanding subtest and 0.52 for the factual knowledge subtest. Note that these reliabilities are low, but given the number of items and the type of questions that are being asked, they are not uncommon. Sinharay (2010) for example, reported an average operational subtest reliability of 0.38 for subtests with an average of 19 items. The PRMSE in estimating the true subtest score from the observed total score (PRMSE_*x*_) was 0.80 for both the conceptual subtest and the factual knowledge subtest. Since the PRMSE_x_ values are larger than the PRMSE*_s_* values, we conclude that reporting subscores would not be useful in this case. In this example the correlation between the conceptual understanding and factual knowledge subtest was 0.54, and the subscores with total score correlations were 0.91 and 0.85 for conceptual understanding and factual knowledge, respectively.

For the statistics exam consisting of open and multiple-choice questions the PRMSE_s_ was 0.63 for the open questions and 0.66 for the multiple choice questions, with a total score reliability of 0.77. Since the PRMSE_x_ was 0.81 for the open questions and 0.84 for the multiple choice questions, both larger than the PRMSE_s_ of both subtests, we conclude that reporting subscores would not be useful for this exam^1^. Furthermore, the correlation between the subtests was 0.63, and the subtest-total test correlation was 0.85 for the open questions and 0.94 for the multiple-choice questions. Note that these results are in agreement with the suggestion made by Sinharay (2010) that subscore-total score correlations larger than 0.85 often result in subscores that do not have added value to the total score. Thus, reporting separate “diagnostic” subscores for the open questions and the multiple choice questions is not useful here.

**FIGURE 1:**
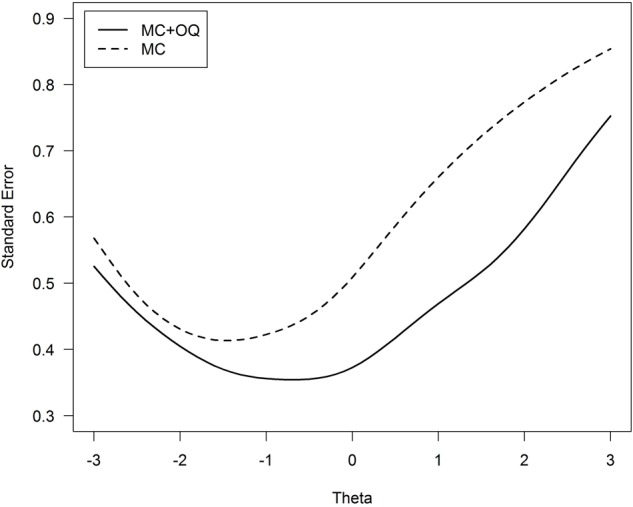
**Standard error of the achievement score (denoted Theta) for the 20 multiple choice items (dashed line) versus 20 multiple choice items plus 5 open ended questions (solid line)**. MC, multiple choice questions; OQ, open-ended questions.

We should remark that this does *not* imply that the open questions do not add to the measurement precision of the total score. We can illustrate this by performing an item response theory analysis (IRT, e.g., Embretson and Reise, 2000) on the data^2^. A nice characteristic of IRT is that it enables us to report the measurement precision (standard error) conditional on an examinee’s score. Interesting is that if we compare the standard error of the examinees’ scores, the open questions reduce this standard error and thus add to the measurement precision of the statistics exam, as shown in **Figure 1**. The test scores are now expressed on a theta metric with a mean of 0 and a SD equal to 1; these theta values are strongly related to the total score (*r* = 0.93). It can be seen that across all achievement levels (theta scores) the combination of multiple choice items and open ended question resulted in a lower standard error than using only multiple choice items, especially at the higher range of the scores.

**FIGURE 2:**
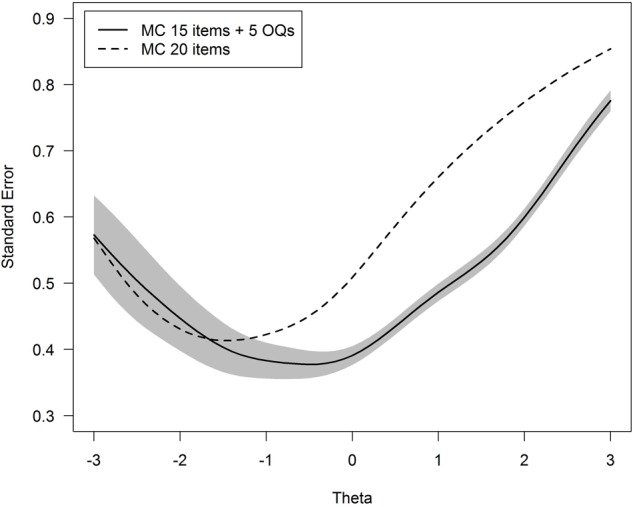
**Standard error of the achievement score (denoted Theta) for the 20 multiple choice items (dashed line) versus the mean of standard error for all tests based on 15 multiple choice items plus 5 open ended questions (solid line), with a 95% variability band around the mean**. MC, multiple choice questions; OQ, open-ended questions.

One could argue that the different number of items between both tests (i.e., 20 multiple choice items versus 25 multiple choice plus open question items) explains the difference displayed in **Figure 1**. We verified that this is not the case by a supplementary simulation analysis. The standard error of theta for all possible tests composed of any 15 multiple choice items plus the 5 open questions was computed, for a total of 15,504 such parallel tests. **Figure 2** shows the mean standard error across the datasets, together with 95% variability bands around the mean value. It can be verified that, for theta values above -1 the standard error based on tests including the open questions are smaller in comparison to the test based on 20 multiple choice items. Thus, the open questions do add to the measurement precision of the total score, in spite of their modest contribution to measuring the true open question subtest scores. This may partly be explained by the partial credit scoring. In general, polytomous scoring increases the test reliability as compared to dichotomous scoring (e.g., Maydeu-Olivares et al., 2009).

A further inspection of the results showed that (i) the inclusion of open questions improved the theta measurement accuracy especially for theta values between 1 and 2 (**Figure 2**), which implies that we are better equipped to measure the score for good students, and (ii) this was in particular the result of two open-ended questions that performed well to discriminate students from each other. These two items were to a large degree responsible for the improved measurement accuracy using the open-ended questions.

## Discussion and Recommendations

Although most researchers and practitioners realize that unreliable subscores should not be used, or should be used with great care, in many publications in educational and psychology studies we often read sentences like “The reliability of the total score equaled 0.80; whereas the reliability of subscore *X* equaled 0.60 and subscore *Y* equaled 0.55.” Then, subtest scores are being used without providing the reader any idea about how useful it is to report these subscores in addition to the total score.

We think that it is very important that teachers and, perhaps especially quality control departments that provide teachers with information about the psychometric quality of test scores, also provide information about the quality of subscores when the latter is considered important. Teachers may indicate which items form a subtest and then control departments may analyze the exam and provide feedback to the teacher. When both total scores and subscores are being reported, a teacher should show that these subscores have added value to the total score because they are interpreted as if they provide information independent from what is also reported in the total score.

Note that in our empirical examples the reliability of the subscores were rather modest, which is not surprising given the small number of items in the total test. What is informative, however, is that the correlations between subscores were modest, suggesting that they can be considered distinct (Sinharay, 2010). When subtests correlate highly, this suggests that the questions of the subtests measure similar things, and that there is a lot of shared variance. Even though the subtest correlations are not very large, however, we found in both exam examples that the estimated reliabilities based on the total tests were much larger than the observed reliabilities of the subtest scores. This was because the correlation between the subtest scores and the total scores was high: 0.85 and 0.94. This means that the subtest scores do not give reliable information about performance on that subtest.

Another important message is that when exams are not *explicitly* constructed to be able to provide scores on subtests, in many cases it will not be possible to use subscores in addition to total scores and report something that we did not already know using the total score. This is an important message for teachers in higher education. We are often inclined to overemphasize the information we can obtain for diagnostic purposes or for formative assessment from subscores on an exam. Thus a first take home message is that as we showed using our empirical examples, using subscores on the basis of standard exams does not necessarily add information to the total score. A second, related take home message is that it will take considerable effort to construct diagnostic exams.

Finally, it was interesting to see that adding a number of open-ended questions to the exam that were scored according to a number of well-described instructions resulted, in general, in more measurement precision than when only using dichotomously scored items. This could be explained by the scoring system: Each open-ended question consisted of a number of dichotomously scored subtasks, thus, in fact, lengthening the test with more than one dichotomously scored “item”. These results are also interesting in the light of the often-found result that open-ended questions do not add to the reliability of a test (see e.g., Hift, 2014). Perhaps when we use a well-described scoring system, these types of open questions may both increase the face validity of an exam and the reliability of an exam, at the expense, of course, of efficiency.

## Author Contributions

RM: Idea and writing. AB: Analysis and writing. CA: Suggestions for a analysis and writing. JT: Analysis. RB: Writing.

## Conflict of Interest Statement

The authors declare that the research was conducted in the absence of any commercial or financial relationships that could be construed as a potential conflict of interest.

## Footnotes

1 We performed a replication study where we used a sample of 318 students who took the same exam in the same year 2014–15, only these were students who followed a Dutch-language program. Thus the exams were the same except for the language. We found very similar results as for the English-language program: PRMSE_s_ = 0.66 and 0.67 for the open questions and the multiple choice subtests, respectively; reliability = 0.78; PRMSE_x_ = 0.79, 0.83; correlation between subtests was 0.62; subtest-total test correlation was 0.86 and 0.94 for the open questions and the multiple choice questions, respectively.

2 We fitted the two-parameter logistic model (e.g., Embretson and Reise, 2000) to the 20 multiple choice items. Furthermore, we fitted the graded response model (Embretson and Reise, 2000) to the five open-ended questions. The scores of the open-ended questions were recoded in terms of the number of correct subtasks per item. This led to scoring four of the five open questions on a five point scale (0, 1, 2, 3, 4) and one open question on a three point scale (0, 1, 2). The IRT models described above were fit to the data by means of the program IRTPRO (version 2.1, Cai et al., 2011) using the default options offered by the software.
