# Corrigendum: The Use of Subscores in Higher Education: When Is This Useful?

**DOI:** 10.3389/fpsyg.2018.00873

**Published:** 2018-05-28

**Authors:** Rob R. Meijer, Anja J. Boevé, Jorge N. Tendeiro, Roel J. Bosker, Casper J. Albers

**Affiliations:** ^1^Faculty of Behavioral and Social Sciences, Psychometrics and Statistics, University of Groningen, Groningen, Netherlands; ^2^Faculty of Behavioral and Social Sciences, Education, University of Groningen, Groningen, Netherlands

**Keywords:** classroom testing, diagnostic testing, formative feedback, test format, subscores, validity open-ended questions

In the original article, there was an error: There were a couple of incorrect numbers.

A correction has been made to the RESULTS section Paragraph 2.

Note that the changes had no further effect on any conclusions.

Sinharay (2010) for example, reported an average operational subtest reliability of 0.38 for subtests with an average of 19 items. The PRMSE in estimating the true subtest score from the observed total score (PRMSE_*x*_) was 0.80 for both the conceptual subtest and the factual knowledge subtest.

The original article has been updated.

## Conflict of interest statement

The authors declare that the research was conducted in the absence of any commercial or financial relationships that could be construed as a potential conflict of interest.

## References

[B1] Sinharay S. (2010). How often do subscores have added value? Results from operational and simulated data. J. Educ. Meas. 47, 150–174. 10.1111/j.1745-3984.2010.00106.x

